# Mind, brain, body, and neuroethics: a conversation with Karen Rommelfanger

**DOI:** 10.1117/1.NPh.9.1.010101

**Published:** 2022-03-28

**Authors:** Anna Devor

## Abstract

Neurophotonics editor-in-chief Anna Devor discusses neuroethics topics related to rapidly advancing neurotechnology, in conversation with Institute of Neuroethics founder Karen Rommelfanger.


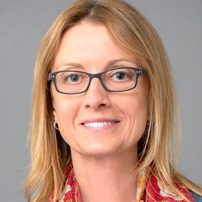
When I was a kid, I thought that white cranes did not exist. In my mind, they were a fantasy, mythological creatures like unicorns, sphinxes, and centaurs. All the cranes I had ever encountered in real life were gray. But I was wrong! White cranes were real, and they looked as fabulous in real life as they were on those mesmerizing paintings and embroideries that once upon a time were my only window into the Far East.

This is what I thought looking through the window of a train in October 2017 on my way to the Global Neuroethics Summit in Daegu, South Korea. My train was making its way across the southern part of the Korean Peninsula, and small rivers and lakes glided by. Totally live and real white cranes stood graciously in shallow waters.

The goal of the Global Neuroethics Summit—supported by the Kavli Foundation and the Korea Brain Initiative—was to convene representative leading scientists and ethicists of brain initiatives and cultural scholars from around the world to discuss cross-cultural perspectives arising at the interface between brain science and societal ethics. During the Summit, we drafted a paper, which was published a year later,^1^ that outlined neuroethics priorities for global brain projects. This is also where I met Dr. Karen Rommelfanger who was co-chairing the Summit with Dr. Sung-Jin Jeong of the Korea Brain Initiative.

**Figure:**
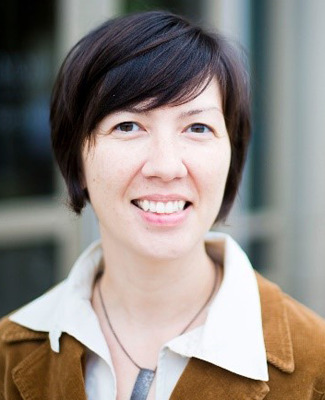
Karen Rommelfanger, Institute of Neuroethics

Dr. Rommelfanger started her academic career as a neuroscientist. For over a decade, she studied Parkinson’s Disease using a broad array of neurotechnologies including brain imaging and electrophysiological recordings. Then she became the founder and director of the Neuroethics Program at Emory University. She was the senior associate editor for the *American Journal of Bioethics Neuroscience* and former executive board member of the International Neuroethics Society. She serves as a member of the BRAIN Initiative’s Neuroethics Working Group and was on the advisory council to the director of the National Institutes of Health (NIH) for BRAIN 2025. In addition, she continues to co-chair the International Brain Initiative’s Global Neuroethics Workgroup and is a member of the Global Futures Council on neurotechnology for the World Economic Forum. During the pandemic she launched a neuroethics consulting firm where she tackles real-world problems for nonprofits, policy entities, and tech startups, by creating and implementing neuroethics strategies in the field. Last fall, she left her full-time faculty position as associate professor at Emory to dedicate her attention to launching an independent think-and-do tank, the Institute of Neuroethics, which is the first international think tank wholly dedicated to neuroethics. I recently asked Dr. Rommelfanger to share her thoughts on several topics related to rapidly advancing neurotechnology in the context of traditionally philosophical values of East and West. Below, I would like to share with you part of this conversation.

*Anna:* Many of our readers are working towards a Doctor of Philosophy (PhD) degree or have it already, but probably not too many know that the original Greek meaning of “philosophy” is “love of wisdom.” You received your PhD in experimental neuroscience before becoming a leader in the field on neuroethics. One of your current areas of research is the ethical implications of emergent brain-machine interfaces (BMI). In your opinion, what does it take to be wise at the interface between neuroscience and neurotechnology?

*Dr. Rommelfanger:* Wisdom is such a loaded word and its meaning across communities and cultures is an entire area of scholarship on its own. But I can speak to the types of qualities one might want to cultivate as a scientist. A graduate neuroscience student who works in my lab, Linzie Taylor, is currently developing a list of points to consider in order to mitigate predominant and often insidious white, western, individualist biases and create more inclusive scientific communities and research outputs. Some of the qualities worth cultivating are reflexivity (examining personal assumptions and worldviews), solidarity and positive reciprocity (exploring shared interests and ensuring the benefits are equitably distributed not just amongst scientists but also for communities), and finally, equality and equity (in not only who enjoys the benefits of research but even which scientists we value and represent in our citations).

*Anna:* Our engineering students start by learning a set of tools followed by how to use these tools and innovate in their development and application. In recent years, there has been an explosion of new neuroengineering tools, such as abovementioned BMI, so naturally there is a lot of excitement about courses that teach what we can do with these tools. There are no engineering courses, however, that teach what we should *not* do. Should there be such courses?

*Dr. Rommelfanger:* When ethical tensions arise in the real world, there’s rarely a black and white answer. Often what I help people do is select the *less* bad option. Often mandatory Responsible Conduct of Research (RCR) training, typically click-based online testing, is pretty bad at teaching critical thinking. And sadly, I believe this kind of training erodes scientists’ ability to understand ethics as a critical tool for creativity and decision-making. Instead, they see ethics as the equivalent of compliance. I often must remind people that just because something is legal doesn’t mean its ethical—consider the history of slavery in the U.S. for example.

Most simply, neuroethics is a field that systematically explores how neuroscience and neurotechnologies impact our value systems as a society and as individuals. Further, neuroethics explores the value conflicts and tensions between neuroscientific discoveries and society.

Many students who are drawn to neuroengineering are curious and driven by the questions about what neuroscience can tell us about human experience and about how neurotechnology could change society. These are ethical questions and worldviews on these issues can shape research design, practice, and dissemination. Students should be empowered with an awareness and basic facility in asking and beginning to systematically address neuroethics questions (Box 1) as part of their scientific toolkit. The scientists of today and tomorrow must be equipped to at a minimum be aware of these questions and considerations and know where to get help to answer them. This could be done through a full course, but I often simply offer a few modules repeatedly throughout undergrad, grad, and postdoc training. I don’t think a single exposure, while it can leave a strong impression, is really enough to empower scientists to be able to integrate systematic ethics analysis into their work.

**Table:** 

Box 1. Neuroethics questions to guide ethical research in the international brain initiatives: NeQN (from Ref. 1).
Q1. What is the potential impact of a biological model or neuroscientific account of disease on individuals, communities, and society?
1a. What are the possible unintended consequences of neuroscience research on social stigma and self-stigma?
1b. Is it possible that social or cultural bias has been introduced in research design or in the interpretation of scientific results?
Q2. What are the ethical standards of biological material and data collection and how do local standards compare to those of global collaborators?
2a. How can human brain data (e.g., images, neural recordings, etc.), and the privacy of participants from whom data is acquired, be protected in case of immediate or legacy use beyond the experiment?
2b. Should special regard be given to the brain tissue and its donors due to the origin of the tissue and its past?
Q3. What is the moral significance of neural systems that are under development in neuroscience research laboratories?
3a. What is the requisite or even minimum features of engineered neural circuitry required to generate a concern about moral significance?
3b. Are the ethical standards for research conduct adequate and appropriate for the evolving methodologies and brain models?
Q4. How could brain interventions impact or reduce autonomy?
4a. What measures can be in place to ensure optimal autonomy and agency for participants/users?
4b. Who will have responsibility for effects (where responsibility has broad meaning encompassing legal, economic, and social contexts)?
Q5. In which contexts might a technology/innovation might be used/deployed?
5a. Which applications might be considered misuse or best uses beyond the laboratory?
5b. Does this research raise different and unique equity concerns and, if so, have equitable access and benefit of stakeholders been considered?

*Anna:* Should the brain be treated differently than other organs? Let’s say that we are bioengineers growing brain or heart organoids in a dish and using photonic tools to image (“read”) and drive (“write”) their electrical activity. Should research on brain organoids be a subject to specific considerations?

*Dr. Rommelfanger:* Science doesn’t exist in a cultural vacuum and the brain has rich cultural meaning in many societies and communities. In studies of predominantly geographically and culturally western communities, the brain is often reported as being synonymous with mind, identity, free-will, decision-making, personhood—the brain is arguably the most self-defining organ in human existence. The brain’s cultural meaning cannot be divorced from biological findings nor vice versa. This is why we see biological information about the brain being used to determine declarations of death, questions on whether entities or individuals are alive. Findings about the brain also impact notions of decision-making and free will and these data have seen their way into courts around the globe. It’s also worth noting that in many cultures, identity and existence are considered from a more relational perspective. For instance, in cultures where philosophical and moral traditions such as Buddhism and Confucianism are embraced, there is a tendency to conceive of the self as relational and not reducible to the brain.

**Figure:**
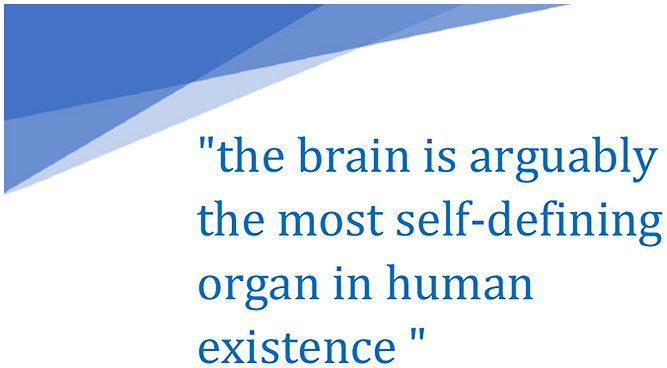


Neural organoid technology is rapidly advancing, and I just attended a workshop a few weeks ago proposing a field called *Organoid Intelligence*. In Japan, scholars have proposed that all human brain organoids be considered “conscious.” Now, should neural organoids be considered to be on equal footing (i.e., equal moral “status”) with nonhuman or human animals? Not necessarily. The better question might be what kinds of moral regard are the human neural organoids owed? In our 2018 paper,^2^ we suggest that researchers consider the moral significance of neural systems that are under development and ask two sub questions. First, what are the requisite of minimum features of engineered neural circuitry required to generate a concern about moral significance amongst a variety of publics. Second, are the ethical standards for research conduct adequate and appropriate for the evolving methodologies and brain models? Importantly, these aren’t questions for just one stakeholder group to answer and act upon.

**Figure:**
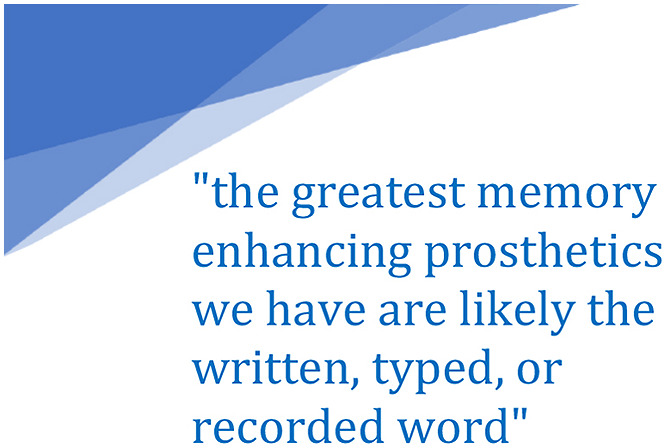


*Anna:* Let’s dwell a little longer on the subject of studying. Wouldn’t it be awesome to be able to “download” knowledge directly into our brains? This concept has been explored, of course, for decades in sci fi movies and literature. But, with the recent push by brain initiatives around the world, we actually start seeing how direct encoding of information into the brain (“brain writing”) one day can become a reality. Wouldn’t that be great for us all? 😊

*Dr. Rommelfanger:* In a recent meeting I attended with the European Parliament’s Science and Technology Options Assessment (STOA) group—a group that considers the long-term impact of emerging technologies for strategic policy making, I was struck that sophisticated, emerging tech policymakers would choose to reference Elon Musk—even amongst all the amazing scientists making breakthroughs today in brain reading and writing. It’s worth saying, and I realize your question is a bit of a tongue-in-cheek question, when considering the ability to download knowledge into our brains, the most sophisticated tool we have is still likely reading or other peripheral modalities (i.e., podcasts and documentaries) for indirectly funneling information into our brains. Similarly, the greatest memory-enhancing prosthetics we have are likely the written, typed, or recorded word.

Cognitive enhancement is one of the favorite neuroethics topics of my students and general public audiences. The ethical considerations vary by context and purpose of the context. Ostensibly, the purpose of education is to not just download information. If we look at the etymology of education, we find the Latin root *educare*, to nourish, and *educere*, to bring out and together, refer to the notion of the educator drawing out, cultivating inner tendencies and resources. If we agree on this conceptualization (and many educators and students may not, in light of the continued corporatization of higher ed), then to simply download information or even memorize information without cultivation of how to use that knowledge would come at odds with the value and purpose of education. Or in other words, might be considered “cheating.” That said, only one U.S. university explicitly notes the use of “study drugs” is considered “cheating” rather than as a violation of the code of conduct via illicit drug use. Of course, there are other concerns related to physical safety, access and fairness, consent, and feeling of pressure to take cognitive enhancers if everyone else is. Some may wonder if making cognitive enhancers available to some versus a few might further exacerbate existing social divides. But, these aspects might be evaluated differently in the context of military use of enhancers (as pilots may be mandatorily expected to use) where physical safety, access, and consent are framed and operationalized differently.

*Anna:* We all love travelling, spending a semester abroad, taking a sabbatical in a collaborator’s lab overseas. Science is a global phenomenon. Linear algebra and differential equations are taught in North America the same way they are taught in Asia or Europe. Do our cultural differences impact our philosophical analysis of neurotechnologies and their effect on the society? If yes, would these differences pose a barrier for international collaboration in some cases?

*Dr. Rommelfanger:* Good neuroethical, neuroscience, and neuroengineering practices should also lead to engaging with the underlying values and ethical concerns that drive brain research across cultures and continents. Ultimately, cultural values influence not only how science is done, but also how the science might be adopted and integrated in societal practice.

It’s important to note that examining cultural differences is not an exercise in looking at the spectacle of the “other” but instead is an opportunity to more deeply explore our own assumptions and world views for greater understanding and collaboration. To be clear, these cultural differences exist between national entities, *within* defined societies, and also *among* individual researchers and practitioners.

Even as the world becomes more globalized, and arguably cross-national or cross-cultural distinctions may be getting smaller, this does not mean that exploration of cultural distinctions should be excluded from neuroethics discourse. Being inclusively oriented means that these distinctions—even if only differing in degree versus kind—can have dramatic impacts on the lives of many.

**Figure:**
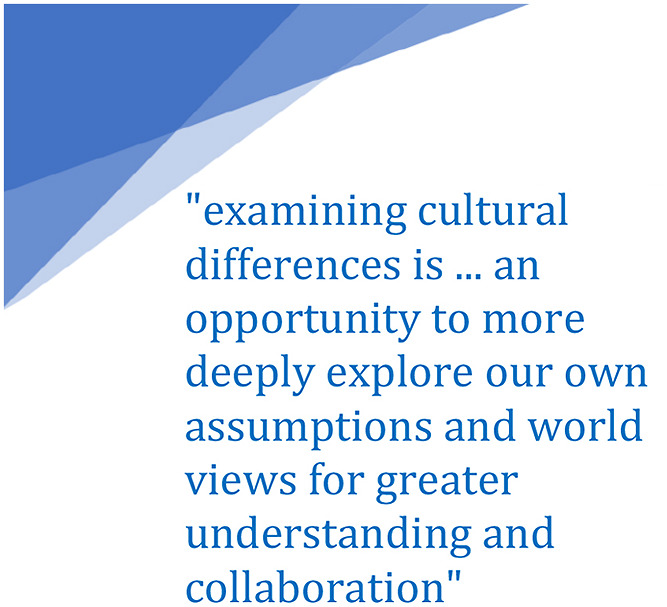


While the brain is culturally special, it’s not necessarily universally special in the same ways or even to the same degree. For example, my research with neurologists and psychiatrists has shown that even today in western medicine, the brain and mind are considered separate entities, having impact on conceptualizations of mental health and stigma. For many cultures, the mind, brain, and body are inextricably linked, as typified by the Chinese and Japanese concept of or *xin* or *kokoro* (⿃), wherein the mind-heart-spirit are inseparable features.

Further, studies have shown that individual views of the association of the essence of self or soul with the brain or the heart influences moral decision-making on issues of personal responsibility (which has implications for law), the moment of conception of life, death, and even human flourishing (such as whether cognitive enhancement is appropriate).

*Anna:* At least in the US, the COVID pandemic has accentuated the need to improve communication between the scientists and the society. We often think about this communication as a one-way street: we make discoveries and develop tools to improve the human condition, and then communicate these advances to the society. Keeping in mind the issues that we just discussed, should this be more of a dialogue when it comes to neurotechnologies?

*Dr. Rommelfanger:* The importance of public dialogue cannot be underestimated particularly with rapidly evolving technologies where the existing and potential impacts on society are so profound. The global pandemic has made it painfully clear how critical ethical-decision-making is with evolving scientific information. The pandemic has also made clear where our gaps are in science communication and public engagement. Even the Organization for Economic Cooperation and Development (a trans-national policy organization) who has created soft law guidance and recommendations for emerging tech, made neurotechnology the first technology they created ethical principles for. One of the key recommendations is that promoting societal deliberation (not just one-way dissemination of knowledge from expert scientists to the public) is critical to responsible innovation with neurotech. Right now, I’m working with them on the implementation phase of this project along with others to explore ways to integrate “neuroethics engagement” into science diplomacy efforts, particularly on the backdrop of rapid proliferation of proposal for governance around neurotechnology. It’s also important to know that the public can provide important insights in considering ethical implications and also informing research design and product development. In a recent IEEE BCI Standards Roadmap, analysis that identified key areas that represented significant gaps in BCI development included lack of end-user insight and input. The scientists of today and tomorrow must be able to engage in some sort of public communication to create a sustainable future for positive neurotechnology development. And luckily there is an entire field that has been systematically developing strategies and methodologies for public engagement in science. Now we just need to promote a culture where scientists are rewarded for doing public engagement work and where the communities of scientists, ethicists, and public engagement specialists can come together. Luckily we’re seeing some support from foundations like The Kavli Foundation and Dana Foundation in these spaces.
